# Combination of thalidomide and *Clostridium butyricum* relieves chemotherapy-induced nausea and vomiting *via* gut microbiota and vagus nerve activity modulation

**DOI:** 10.3389/fimmu.2023.1220165

**Published:** 2023-06-22

**Authors:** Xuanqi Zhao, Heng Wu, Ruizhe Zhu, Gaishuang Shang, Jing Wei, Haitao Shang, Puyuan Tian, Tingtao Chen, Hong Wei

**Affiliations:** ^1^ Precision Medicine Institute, The First Affiliated Hospital, Sun Yat-sen University, Guangzhou, China; ^2^ National Engineering Research Center for Bioengineering Drugs and the Technologies, Institute of Translational Medicine, Nanchang University, Nanchang, China; ^3^ Eastsea Pharma Co. LTD, Qingdao, China

**Keywords:** CINV, thalidomide, intestinal microecology, *Clostridium butyricum*, gut- brain axis

## Abstract

Nausea and vomiting (CINV) are distressful and widespread side effects of chemotherapy, and additional efficient regimens to alleviate CINV are urgently needed. In the present study, colorectal cancer (CRC) mice model induced by Azoxymethane (AOM)/Dextran Sodium Sulfate (DSS) was employed to evaluate the cancer suppression and CINV amelioration effect of the combination of thalidomide (THD) and *Clostridium butyricum*. Our results suggested that the combination of THD and *C. butyricum* abundantly enhanced the anticancer effect of cisplatin *via* activating the caspase-3 apoptosis pathway, and also ameliorated CINV *via* inhibiting the neurotransmitter (e.g., 5-HT and tachykinin 1) and its receptor (e.g., 5-HT_3_R and NK-1R) in brain and colon. Additionally, the combination of THD and *C. butyricum* reversed the gut dysbacteriosis in CRC mice by increasing the abundance of *Clostridium*, *Lactobacillus*, *Bifidobacterium*, and *Ruminococcus* at the genus level, and also led to increased expression of occludin and Trek1 in the colon, while decreased expression of TLR4, MyD88, NF-κB, and HDAC1, as well as the mRNA level of IL-6, IL-1β, and TNF-α. In all, these results suggest that the combination of THD and *C. butyricum* had good efficacy in enhancing cancer treatments and ameliorating CINV, which thus provides a more effective strategy for the treatment of CRC.

## 1 Introduction

Colorectal cancer (CRC) is a cancer occurring in the colon or rectum, with over 1.9 million new cases and 935,000 deaths estimated in 2020, ranking the third in incidence and the second in mortality (1, 2). For patients’ treatment, systemic chemotherapy is the mainstay treatment besides surgery and local radiotherapy and has been wildly used in advanced CRC (3). Currently, platinum-based chemotherapy drugs which inhibits nuclear DNA transcription and replication and initiates programmed cell death are dominating (4). It is proposed that cyclooxygenase-2 (COX-2) inhibition can hasten Caspase-3 activation and poly (ADP-ribose) polymerase (PARP) cleavage, as well as suppress the expressions of anti-apoptotic proteins such as surviving, thus activating tumor apoptosis (5).

Despite the anticancer effects, chemotherapy drugs lead to severe side effect include gastrointestinal toxicities, myelosuppression, immunosuppression and neurotoxicity due to poor targeting (6, 7). Chemotherapy-induced nausea and vomiting (CINV) is a common adverse effects of chemotherapy that affects patient’s life and treatment effectiveness (8, 9). It has been reported that the expression of 5-hydroxytryptamine (5-HT) in intestinal mucosa was triggered by cytotoxic chemotherapy drugs, then stimulating the 5-HT receptors on adjacent vagal afferent nerves (VAN) (10). After nerves depolarization, vomiting center in brainstem was stimulated to induce a vomiting reflex (11). Consequently, there is a pressing need for alternative treatments that are both highly effective and have minimal side effects.

The most commonly used antiemetic agents belong to neurokinin-1 receptor antagonists (NK_1_-RAs), 5-hydroxytryptamine-3 receptor antagonists (5HT_3_-RAs) and dexamethasone but the therapeutic effect was still not satisfactory (12, 13). Thalidomide (THD), a derivative of glutamate, was originally used to treat nausea in pregnancy, later was banned because of its teratogenicity (14, 15). However, THD, as approved drugs, has been used in the treatment of a variety of solid tumors, with good efficacy and a certain degree of safety (16). At present, the effectiveness of THD in controlling CINV has become the focus of research. The randomized control trials have clinically confirmed the effectiveness of THD by calculating and analyzing the delayed and overall complete response rates to vomiting in cancer patients (17). Subsequently, several studies have demonstrated that THD’s potent immunomodulatory activity affects the expression and activity of various cytokines to influence anti-angiogenic effects and tumor defense (18, 19). However, numerous studies have shown that patients with CRC have an altered intestinal microbiota which lost a significant amount of butyrate-producing bacteria, such as *Clostridium*, *Roseburia*, and *Eubacterium* spp. (20). Chemotherapeutic drugs disrupt intestinal microecology, damage intestinal mucosal barrier, and trigger gut inflammation (21). Furthermore, the use of THD will lead to gastrointestinal side effects including constipation due to the certain neurotoxicity of THD (22).

At present, the gut microbiota has been widely evidenced to be related with the occurrence, development and treatment of cancers (23). Probiotics are beneficial microorganisms exerting probiotic role by regulating the gut microbiota (24, 25). *Clostridium butyricum* generates short-chain fatty acids (SCFAs), especially butyrate and acetate, and affects various physiological processes contributing to host health (26, 27). *C. butyricum* CBM 588 has been implicated in anti-inflammation, gut epithelial barrier protection and the increased abundance of *Lactobacillus*, *Bifidobacterium* in the gut microbiota (28). A study conducted by Danfeng Chen et al. revealed that *C. butyricum* can inhibit the development of CRC by the stimulation of apoptosis and the reconstruction of gut microbiota (29). Moreover, *C. butyricum* administration was found to significantly reduce cognitive dysfunction and histopathological changes, as well as neuronal apoptosis probably *via the* involvement in gut-brain axis modulation (30). Therefore, this suggests a possible therapeutic role for *C. butyricum* in the gastrointestinal toxic side effects of chemotherapy.

In clinic, we found that the combination of *C. butyricum* and THD almost eliminate CINV (data unshown). Therefore, to elucidate the efficacy and mechanisms of *C. butyricum*+THD on treatment of CINV, we investigated the antitumor function, the preventing of nausea and vomiting, the altering of microbiota composition and the enhancement of the intestinal epithelial barrier in CRC mouse model with chemotherapy. This study presents new ideas for the prevention and treatment of CINV with *C. butyricum*+THD.

## 2 Materials and methods

### 2.1 Animals and experimental design

**Figure 1:**
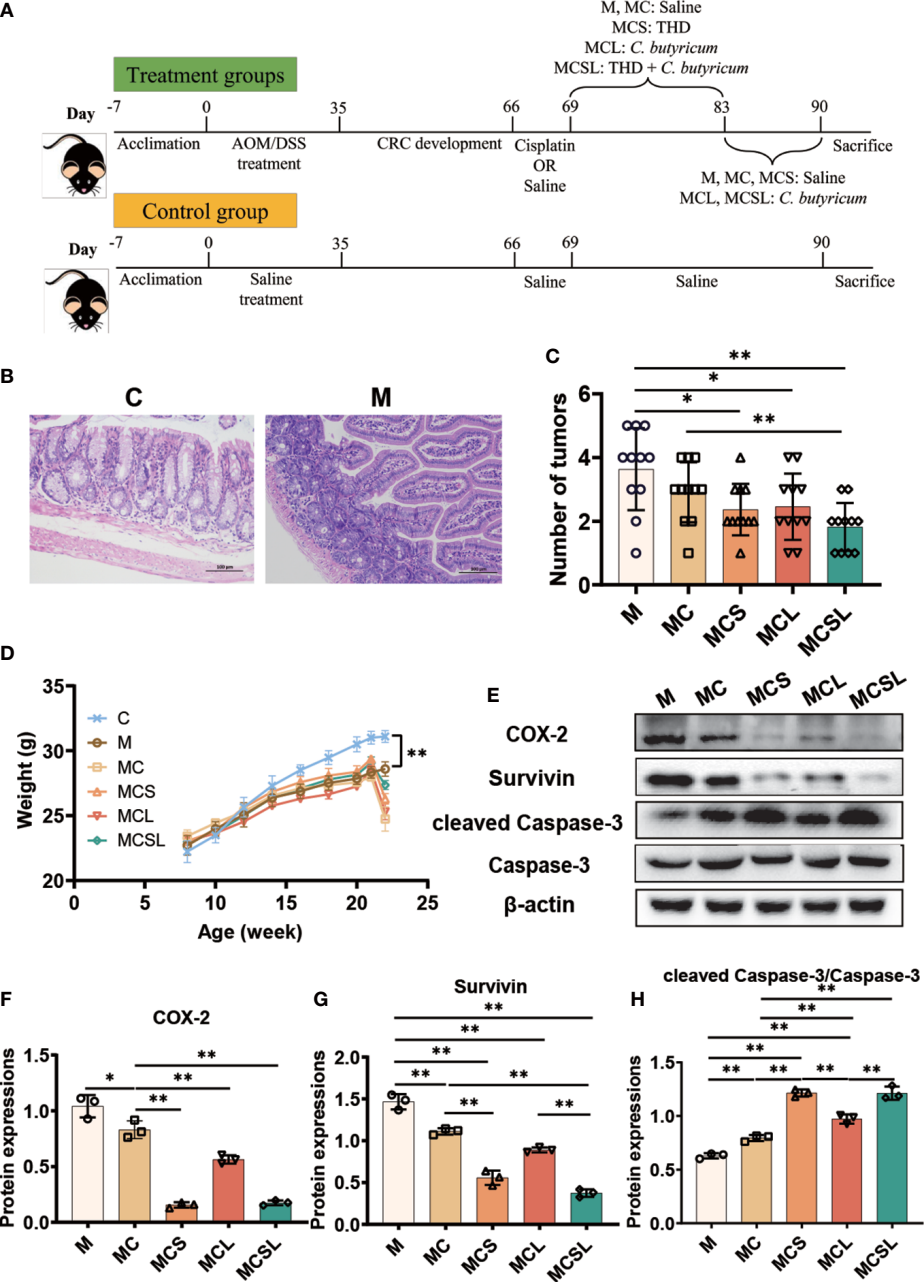
The combination of THD and *C*. *butyricum* promoted tumor apoptosis in CRC mice. **(A)** The schematic diagram of CRC modeling and treatment process in C57BL/6 mice. **(B)** H&E staining of colon tissue. **(C)** The number of tumors in colon tissue (n=11). **(D)** Weight of mice in different treatment groups (n=11). **(E)** Western blotting of COX-2, Survivin, cleaved Caspase-3 and Caspase-3 (n=3). **(F–H)** Relative expressions of COX-2, Survivin and cleaved Caspase-3/Caspase-3 (n=3). Significance determined using one-way ANOVA with Tukey’s multiple comparison test and expressed as mean ± SD, *P < 0.05, **P < 0.01.

Seventy-two male C57BL/6 mice were obtained from Hunan SJA Laboratory Animal Co., Ltd. (Changsha, Hunan, China) and housed in an animal facility with a standard 12 h light–dark cycle. **Figure 1A** depicts the animal treatment schedule. After 1 week acclimation, the mice were randomly assigned to six different groups including: (i) C group, a control group with saline intraperitoneal injection; (ii) M group, a model group with injected Azoxymethane (AOM) (MP Biomedicals LLC, Santa Ana, America, 183971) (10 mg/kg) twice at the 1^st^ and 19^th^ day and fed 1% Dextran Sodium Sulfate (DSS) (Meilunbio, Dalian, China, MB5535) three times for 6 days each time at the 7^th^,19^th^ and 29^st^ day; (iii) MC group, treated with AOM/DSS, then received 2.5 mg/kg cisplatin (Macklin, Shanghai, China, D807330) after 1 month *via* intraperitoneal injection for 3 consecutive days; (iv) MCS group, after AOM/DSS and cisplatin treatment, treated with 25 mg/kg THD (Ark Pharm, Chicago, America, AK-91024) *via* intraperitoneal injection for 14 consecutive days; (v) MCL group, after AOM/DSS and cisplatin treatment, treated by gavage with 10^7^ CFU *C. butyricum* for 21 consecutive days; (vi) MCSL group, after AOM/DSS and cisplatin treatment, treated both 10^7^ CFU *C. butyricum* for 21 consecutive days and 25 mg/kg THD for 14 consecutive days.

### 2.2 Behavioral experiments

Kaolin intake measurement was performed for 3 consecutive days during chemotherapy. Kaolin powder was prepared by mixing kaolin (Macklin, Shanghai, China, K812212) and Arabic gum (Meilunbio, Dalian, China, MB1728) at a 99:1 ratio and provided to mice. After drug intervention, the amount and residual of chow intake and kaolin intake were measured after drying every 24 h, and the ratio of kaolin intake to total food intake was calculated.

### 2.3 Sample collection

After stopping administration, fecal samples were collected and the mice were euthanized with isoflurane gas anesthesia. Colon, tumor, and brain tissues were immediately collected, fixed, or stored at -80 °C.

### 2.4 Histological staining analysis

The fixed colon and brain tissue samples were embedded in paraffin, cut into 2-μm-thick sections, and possessed with H&E staining to evaluate microscopically. And immunohistochemistry staining was possessed by primary antibody incubation (**Supplementary Material Table S1**), washing, and corresponding secondary antibody incubation.

### 2.5 ELISA

Brain and colon tissues were homogenized and then the supernatant was collected for ELISA. According to the instructions of the ELISA kit (mlbio, Shanghai, China, ml001891), the protein expression of 5-HT was measured and determined at 450 nm with the microplate reader.

### 2.6 Quantitative real-time PCR

Total RNA was prepared from brain and colon tissues, reverse transcribed to cDNA and conducted forty cycles as follows: 95°C for 30 s, 60°C for 30 s, followed by 60 s at 95°C for polymerase activation using corresponding primers (**Supplementary Material Table S2**).

### 2.7 Immunoblotting

Tumor and colon tissues were homogenized and the supernatant was collected for western blotting. Total proteins were fractionated with SDS–PAGE and transferred to the PVDF membrane. After blocking, staining with primary antibodies (**Supplementary Material Table S1**), and incubating with HRP-conjugated secondary antibodies, the membranes were visualized by an ECL system.

### 2.8 High-throughput sequencing analysis

Total genome DNA from mouse feces was extracted, then the V4 hypervariable regions of 16S rDNA were amplified by PCR. After sequencing the PCR amplification products and quality filtering, the reads were clustered as OTU using VSEARCH clustering (v2.13.4_linux_x86_64) sequence. Based on the OTU profiles, observed species, alpha diversity (Shannon), and beta diversity (PCoA) were calculated. Venn Diagram was used to determine the relationships between communities among different treatment groups.

### 2.9 Statistical analysis

All data are presented as mean ± standard deviation. Statistical evaluation was performed using one-way ANOVA followed by Tukey’s multiple comparison test as *post hoc* tests *via* GraphPad Prism 9.0 software at *P* value of < 0.05 (*) or < 0.01 (**).

## 3 Results

### 3.1 The combination of THD and *C. butyricum* promoted tumor apoptosis in CRC mice

To verify the construction of the CRC mouse model, the colon of mice was collected for H&E staining after the AOM/DSS treatment. Compared to the C group, M group showed an increasing number of submucosal glands having dysplasia into the lumen and detachment of intestinal mucosal villi in colon, the enlarged and deeply stained cell nuclei, and mitotic figures, which confirmed the successful construction of the CRC mouse model (**Figure 1B**).

Then we treated the CRC mice with different therapies. In our study, cisplatin treatment reduced the colon tumor number of mice than M group (M vs. MC = 3.64 vs. 2.91, **Figure 1C**), while the ingestion of THD (2.36, *p* < 0.05) and *C. butyricum* (2.45, *p* < 0.05) further promoted the anti-tumor effect. Notably, the combination of THD and *C. butyricum*-treated mice in MCSL group had markedly fewer colon tumor numbers than MC group (MC vs. MCSL =2.91 vs. 1.82, **Figure 1C**, *p* < 0.01). In addition, the body weight of CRC mice was significantly lower than that in C group (C vs. M = 31.10 vs. 28.60, **Figure 1D**, *p* < 0.01), which was aggravated after cisplatin treatment in MC group (24.76, *p* < 0.01) and was alleviated in MCS group (26.30, *p* < 0.01), MCL group (25.31, *p* < 0.01) and MCSL group (27.36, *p* < 0.01), indicating that the survival state of CRC mice was improved.

Furthermore, western blotting of tumor certified the decreased activity of COX-2 (*p* < 0.05) and Survivin (*p* < 0.01), and the increased activity of cleaved Caspase-3 (*p* < 0.01) in MC group than M group, which were remarkedly reversed in MCSL group (*p* < 0.01) (**Figures 1E–H**). What’s more, the expression of cleaved PARP (p < 0.01) was also increased after the combination of THD *and C. butyricum* (**Figure S1**). Therefore, results indicated that combination therapy of THD *and C. butyricum* optimally enhanced the activation of Caspase-3 apoptotic pathway by cisplatin.

### 3.2 The combination of THD and *C. butyricum* reduced nausea and vomiting after chemotherapy in CRC mice

**Figure 2:**
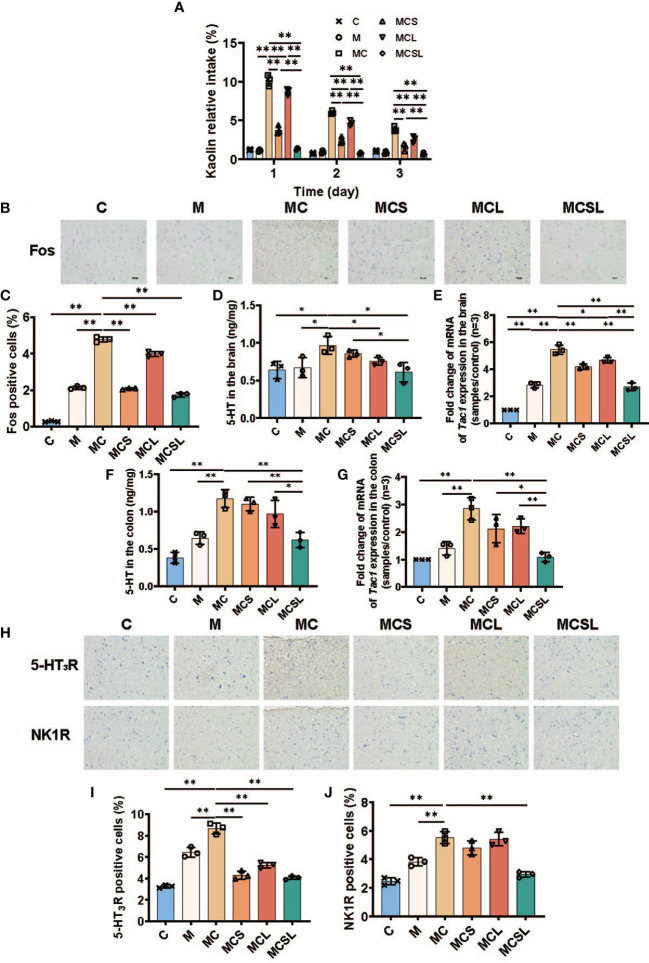
The combination of THD and *C. butyricum* reduced CINV in CRC mice. **(A)** The kaolin consumption of mice in different treatment groups (n=3). **(B)** Fos protein in brain by IHC staining. **(C)** Fos positive cells were semiquantitatively assessed (n=3). **(D)** 5-HT of brain in different treatment groups (n=3). **(E)** the mRNA levels of *Tac1* in brain (n=3). **(F)** 5-HT of colon in different treatment groups (n=3). **(G)** the mRNA levels of *Tac1* in colon (n=3). **(H)** 5-HT_3_R and NK1R protein in brain by IHC staining. **(I, J)** 5-HT_3_R **(I)** and NK1R **(J)** positive cells were semiquantitatively assessed (n=3). Significance determined using one-way ANOVA with Tukey’s multiple comparison test and expressed as mean ± SD, *P < 0.05, **P < 0.01.

Since chemotherapeutic agents are closely connected with the occurrence of nausea and vomiting (31), we sought to explore whether *C. butyricum* and THD treatment could improve nausea and vomiting after chemotherapy. During the kaolin intake measurement, the ingestion of kaolin in each mice group is shown in **Figure 2A**, which is seen as a phenomenon similar to nausea and vomiting in humans.

During the assay, the kaolin intake of MC group on the 1^st^ day was significantly increased than M group (M vs. MC = 1.10% vs. 10.17%, **Figure 2A**, *p* < 0.01). Moreover, compared with MC group, nausea and vomiting was markedly alleviated in MCL group (8.72%, *p* < 0.01), MCS group (3.77%, *p* < 0.01), and MCSL group (1.35%, *p* < 0.01), respectively. Over the next two days, the combination of THD and *C. butyricum* still showed a best therapeutic effect on nausea and vomiting after chemotherapy than MCS group (*p* < 0.01) and MCL group (*p* < 0.01).

To further elucidate the mechanisms in regulation of CINV, we detected the expression of Fos in area postrema (AP) using immunohistochemical staining. In our research, compared to the C group, the expression of Fos in AP of MC group was increased (C vs. MC = 0.28% vs. 4.78%, **Figure 2C**, *p* < 0.01), while both THD (2.08%, *p* < 0.01), *C. butyricum* (3.98%, *p* < 0.01) and the combination treatment (1.74%, *p* < 0.01) could reduce the expression of Fos (**Figures 2B, C**). Notably, the combination of THD and *C. butyricum* treatment completely eliminated Fos expression in mouse brain of MCSL group.

5-HT and tachykinin 1 (Tac1), neurochemical mediators in the brainstem, are associated with emesis caused by cisplatin (31). Therefore, we detected the expression of 5-HT and *Tac1* mRNA in mice brains by ELISA and q-PCR, respectively. The expression of 5-HT (M vs. MC = 0.67 ng/mg vs. 0.97 ng/mg, **Figure 2D**, *p* < 0.05) in mice brains was significantly increased after cisplatin treatment, which was reduced after the combined therapy of THD and *C. butyricum* (MC vs. MCSL = 0.97 ng/mg vs. 0.61 ng/mg, *p* < 0.05). Consistently, *Tac1* mRNA in mice brains was markedly upregulated in MC group (M vs. MC = 2.84 vs. 5.45, **Figure 2E**, *p* < 0.01) and this increase could be reversed in MCS group (4.20, *p* < 0.01), MCL group (4.65, *p* < 0.05) and MCSL group (2.70, *p* < 0.01).

Furthermore, cisplatin has been shown to evoke the release of 5-HT and Tac1, which lead to the activation of a vomiting reflex (11). The content of 5-HT was quantified by ELISA of colon tissue to found that it in MC group was significantly raised than M group (M vs. MC = 0.65 ng/mg vs. 1.17 ng/mg, **Figure 2F**, *p* < 0.01), which was substantially decreased by combined administration in MCSL group (0.62 ng/mg, *p* < 0.01). In addition, *Tac1* mRNA in mice colon was increased after cisplatin injection (M vs. MC = 1.41 vs. 2.84, **Figure 2G**, *p* < 0.01), and the combination of THD and *C. butyricum* could notably downregulate the relative expression of *Tac1* mRNA in CRC mouse with cisplatin chemotherapy (1.09, *p* < 0.01).

5-HT and Tac1 stimulate its receptors (5-HT_3_R and NK-1R) on VAN to stimulate emesis (32). Hence, we further investigated the amount of 5-HT_3_R and NK-1R in AP using immunohistochemical staining. Compared to M group, cisplatin chemotherapy led to abnormal aggregation of 5-HT_3_R (M vs. MC = 6.46% vs. 8.66%, **Figure 2I**, *p* < 0.01) and NK-1R (M vs. MC = 3.83% vs. 5.51%, **Figure 2J**, *p* < 0.01), however, it was significantly reduced in MCSL group (4.07%, **Figure 2I**, *p* < 0.01; 2.93%, **Figure 2J**, *p* < 0.01) (**Figures 2H–J**).

### 3.3 The combination of THD and *C. butyricum* reversed dysbacteriosis in CRC mice

**Figure 3:**
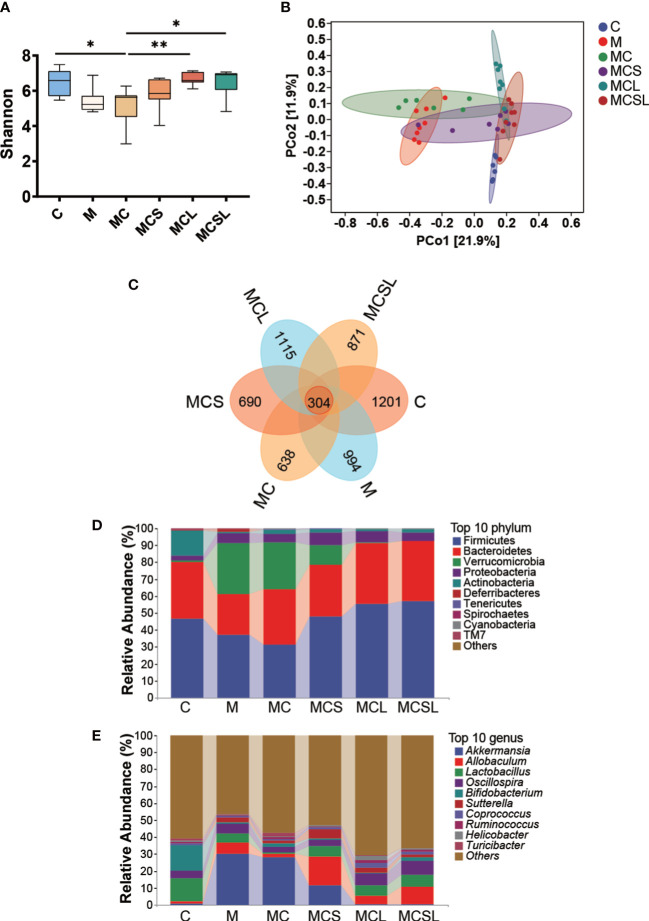
The combination of THD and *C. butyricum* reversed dysbacteriosis in CRC mice. **(A)** The Shannon indexes. **(B)** Principal coordinate analysis (PCoA). **(C)** Venn representation. **(D)** The relative abundance of the bacteria at phylum level. **(E)** The relative abundance of the bacteria at genus level. *P < 0.05, **P < 0.01.

Since disturbed intestinal microbiota is associated with CRC and cisplatin chemotherapy (33), we further analyzed intestinal microbial composition. The Shannon index was markedly reduced in MC group (*p* < 0.05) than C group, and this reduction was ameliorated after *C. butyricum* treatment (MCL group) (*p* < 0.01) (**Figure 3A**). PCoA plots showed that the samples of M group clustered separately from those of C group. Compared to M group, the dysbacteriosis was further exacerbated after cisplatin chemotherapy (MC group), however, it was reversed by the combined treatment of THD and *C. butyricum* (**Figure 3B**). Next, according to the Venn results, there are 304 core OTUs in all of these fix groups, and 1201, 994, 638, 690, 1115 and 871 unique OTUs discovered in C, M, MC, MCS, MCL and MCSL group, separately (**Figure 3C**).

At the phylum level, a lower abundance of Firmicutes, Bacteroidetes, and Actinobacteria, and an increasing amount of Verrucomicobiota were detected in M and MC group compared to C group. After being treated with *C. butyricum*, the relative abundance of the aforementioned phyla significantly recovered (**Figure 3D**). At the genus level, we found that *Akkermansia* was abnormally increased and *Bifidobacterium* and *Lactobacillus* were reduced in feces of M group and MC group than C group. After *C. butyricum* treatment, dysbiosis of these genera was substantially restored than MC group (**Figure 3E**).

**Figure 4:**
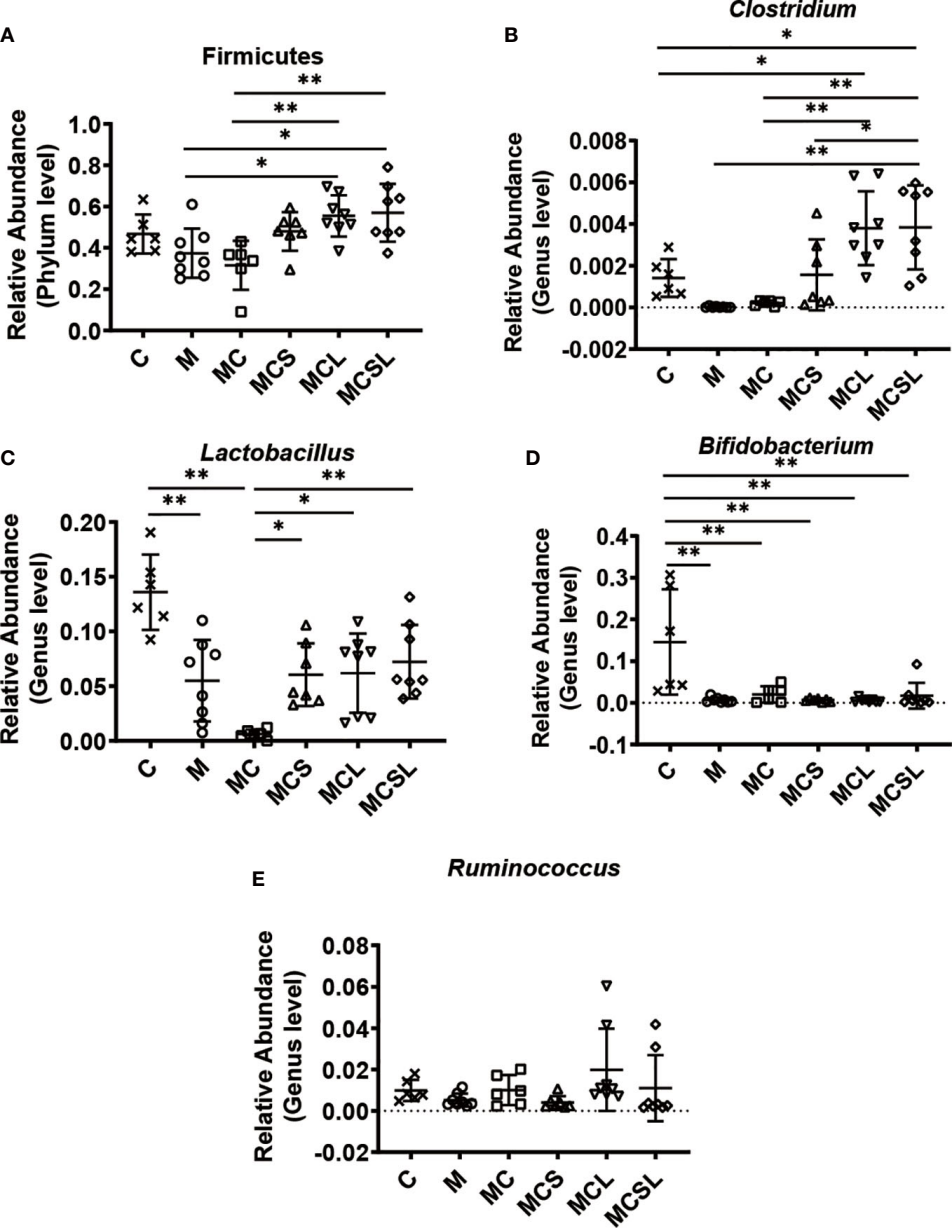
The combination of THD and *C. butyricum* regulated anti-cancer related probiotics in CRC mice. **(A)** The relative abundance of Fimicutes (n=6). **(B)** The relative abundance of *Clotridium* (n=6). **(C)** The relative abundance of *Lactobacillus* (n=6). **(D)** The relative abundance of *Bifidobacterium* (n=6). **(E)** The relative abundance of *Ruminococcus* (n=6). Significance determined using one-way ANOVA with Tukey’s multiple comparison test and expressed as mean ± SD, *P < 0.05, **P < 0.01.

Furthermore, we selected some representative probiotics closely related to CRC and cisplatin-induced vomiting for analysis. Compared with MC group, the relative abundance of the phylum Firmicutes and the genera *Clostridium* was increased in MCL group (*p* < 0.01) and MCSL group (*p* < 0.01) (**Figures 4A, B**). After cisplatin chemotherapy, the relative abundance of the genera *Lactobacillus* was significantly reduced in MC group (*p* < 0.01), while it was markedly increased in MCS group (*p* < 0.05), MCL group (*p* < 0.05), and MCSL group (*p* < 0.01) (**Figure 4C**). Then, we found that the relative abundance of the genera *Bifidobacterium* was greatly reduced after all treatments by comparing with control group (*p* < 0.01), whereas it was slightly elevated in MCSL group (**Figure 4D**). Besides, compared with MCS group, the relative abundance of the genera *Ruminococcus* were up-regulated in MCL group and MCSL group (**Figure 4E**). These results suggested that the composition of gut microbiota could be altered after incidence of CRC and cisplatin treatment, but the use of *C. butyricum* restored the imbalance of the microbiome to a standard one.

### 3.4 The combination of THD and *C. butyricum* inhibited colon inflammation and enhanced intestinal barrier

**Figure 5:**
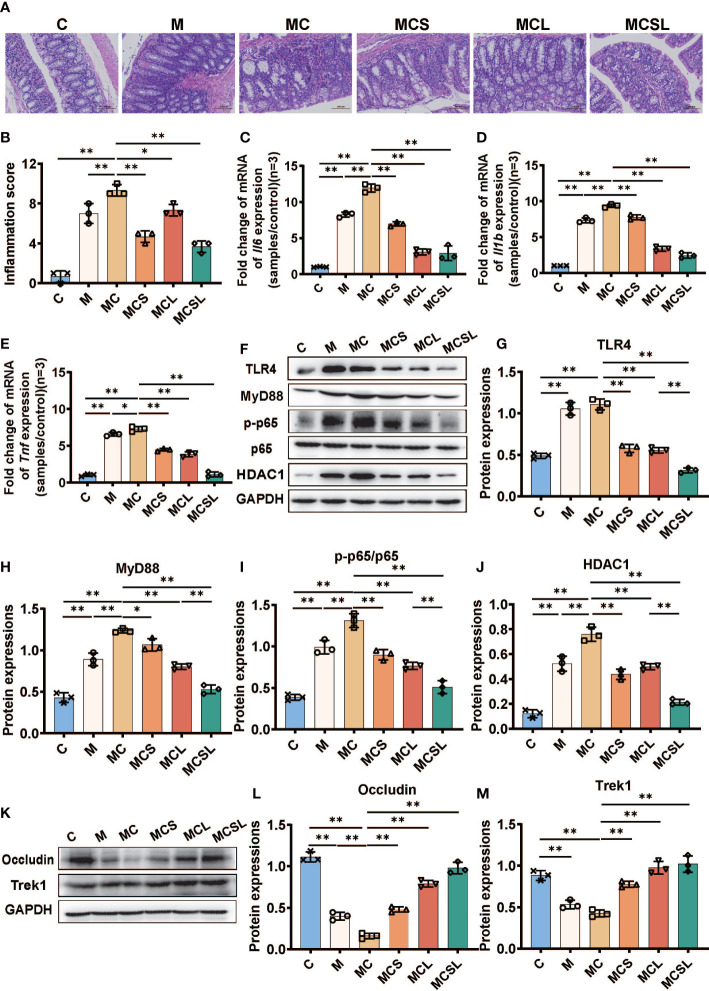
The combination of THD and *C. butyricum* inhibited colon inflammation and improved intestinal barrier in AOM/DSS-induced CRC mice. **(A)** H&E staining of colon tissue. **(B)** Histological scores of inflammations (H&E staining) (n=3). **(C–E)** The mRNA levels of *Il6*, *Il1b*, and *Tnf* (n=3). **(F)** Western blotting of TLR4, MyD88, p-p65, p65, HDAC1 (n=3). **(G–J)** The relative abundance of TLR4, MyD88, p-p65/p65, HDAC1 (n=3). **(K)** Western blotting of occludin and Trek1 (n=3). **(L, M)** The relative abundance of occludin and Trek1 (n=3). Significance determined using one-way ANOVA with Tukey’s multiple comparison test and expressed as mean ± SD, *P < 0.05, **P < 0.01.

Cisplatin will lead to gastrointestinal side effects including colon inflammation and intestinal barrier destruction (34). Hence, we detected colonic inflammation at the end of experiment and the pathological changes of colon showed that the colonic crypts disappearance and inflammatory cell infiltration in M and MC group was significantly alleviated after the combination treatment of THD and *C. butyricum* in MCSL group (**Figures 5A, B**). After that, we performed the qRT-PCR of the intestinal tissue to access the level of inflammatory cytokines. Compared with C group, the mRNA expression of *Il6* (C vs. M = 1.00 vs. 8.24, **Figure 5C**, *p* < 0.01), *Il1b* (C vs. M = 1.00 vs. 7.33, **Figure 5D**, *p* < 0.01) and *Tnf* (C vs. M = 1.01 vs. 6.57, **Figure 5E**, *p* < 0.01) in CRC mice were significantly elevated. Cisplatin chemotherapy in MC group would further upregulate the relative expression of *Il6* (11.92, *p* < 0.01), *Il1b* (9.36, *p* < 0.01) and *Tnf* (7.28, *p* < 0.05) than M group, whereas THD (*p* < 0.01), *C. butyricum* (*p* < 0.01), and the combination of THD and *C. butyricum* (*p* < 0.01) inhibited the up-regulated levels of these inflammatory cytokines in CRC mice with cisplatin chemotherapy.

Then, we further determined HDAC1 protein, key proteins of TLR4/NF-κB inflammatory pathway, and intestinal tight-junction associated proteins in colon by Western blotting. In contrast to normal mice, we detected a marked up-expression of TLR4 (*p* < 0.01), MyD88 (*p* < 0.01), p-p65 (*p* < 0.01), and HDAC1 (*p* < 0.01) in CRC mice. What’s more, cisplatin treatment further aggravated the quantity of MyD88 (*p* < 0.01), p-p65 (*p* < 0.01), and HDAC1 (*p* < 0.01) than M group. On the contrary, the colon inflammation could be suppressed after THD and *C. butyricum* administration (*p* < 0.01) (**Figures 5F–J**).

Meanwhile, Western blotting also revealed the decreased expression of occludin (*p* < 0.01) and Trek1 (*p* < 0.01) in M group than C group, while these reductions were markedly restored in MCS group (*p* < 0.01), MCL group (*p* < 0.01), and MCSL group (*p* < 0.01) (**Figures 5K–M**).

## 4 Discussion

CRC is the third most common cancer worldwide, with high morbidity and mortality rates (35). Currently, chemotherapy, the primary treatment for advanced CRC, causes severe side effects, especially nausea and vomiting (31). CINV which threatens patient’s life and health (8, 9), leads to an urgent need to create alternative therapies that are highly effective and have minimal side effects.

Clinically, we observed that the combination of *C. butyricum* and THD almost eliminate CINV (data not shown), but the mechanism for the prevention of CINV remains unclear. Herein, we used AOM/DSS-induced CRC mouse models that received cisplatin chemotherapy to investigate the primary role of the combination of *C. butyricum* and THD in addition to its possible therapeutic mechanism. Our results indicated that AOM/DSS caused the occurrence of CRC in mice, but the mice receiving cisplatin with the combination of THD and *C. butyricum* showed a markedly decreasing tumor number. Our findings suggested that COX-2 and survivin in tumor are highly expressed, and cleaved Caspase-3 and cleaved PARP are lowly expressed in M group. Additionally, COX-2, which is elevated expressed in CRC, contributes to tumorigenesis by excited angiogenesis, suppressed apoptosis, and enhanced cell invasiveness (36). What’s more, survivin is an inhibitor of programmed cell death that is negatively correlated with apoptosis and directly suppresses caspase-3 activity to prevent apoptosis (37, 38). After activation of caspase-3, the DNA repair enzyme PARP was cleaved leading to loss of the ability of DNA repair resulting in apoptosis (39). Herein, we found that the combination of THD and *C. butyricum* can inhibit the expression of COX-2 and survivin to activate Caspase-3 and cleave PARP to induce tumor apoptosis (**Figure 1**).

Cisplatin, a chemotherapeutic agent frequently used in clinical practice, is associated with emesis (40). Hence, we used kaolin intake measurement, an index of nausea and emesis in animal studies (41), to evaluate nausea and vomiting of mice with chemotherapy. Mice ingested cisplatin severely vomited than M group, but the combination therapy of THD and *C. butyricum* significantly relieved emesis. Fos in AP, a marker of neuronal activity whose expression correlates with brain stimulation resulting from the stimulation of neurotransmitter receptors on adjacent vagal afferents, was increased after cisplatin chemotherapy (42, 43). Our findings suggested that the combination of THD and *C. butyricum* could abundantly decrease the fos expression in the AP, confirming reduced brain neuron activation. Furthermore, it was currently reported that cisplatin-induced release of the 5-HT and Tac1 interacted with receptors (i.e., 5-HT_3_R and NK-1R) of the vagus nerve in brain and gastrointestinal tract to cause the above neuronal activation (7, 44). We found that a combination of THD and *C. butyricum* abundantly reduces the 5-HT expression and the relative *Tac1* mRNA level in brain and colon compared with MC group. Additionally, we found that the combination therapy inhibited aggregation of 5-HT_3_R and NK-1R in brain of CRC mice with cisplatin chemotherapy. In summary, these results suggested that the combined administration of THD and *C. butyricum* reversed the up-release of neurotransmitters and activation of receptors in cisplatin-treated CRC mice model, to relieve the cisplatin-induced nausea and vomiting (**Figure 2**).

Disordered gut microbiota is associated with weakened health (45) and is involved in gastrointestinal carcinogenesis, while cisplatin chemotherapy further destroyed its homeostasis (46, 47). In addition, Chen et al. discovered that *C. butyricum* is beneficial in the reconstruction of gut microbiota (48), and the reversed gut microbiota disorder further plays a role in treatment of intestinal diseases (49). Therefore, we explored whether *C. butyricum* could improve the intestinal microbiota disorder caused by CRC and chemotherapy *via* high-throughput sequencing. The results demonstrated the therapeutics of the combination treatment (MCSL group) in restoration of intestinal microbiota diversity (**Figure 3**). Moreover, it was observed that certain genera of bacteria had reduced abundance in both M group and MC group, including *Clostridium*, *Lactobacillus*, *Bifidobacterium* and, *Ruminococcus*. Furthermore, such dysbiosis could be restored by combined treatment with THD and *C. butyricum*, which is consistent with the published reports about the microbiota regulatory effect of *C. butyricum and* (**Figures 4B–E**). Among them, the increased relative abundance of *Clostridium* may be related to the well intestinal colonization of *C. butyricum* (50). After its colonization, *C. butyricum* furthur regulates the richness and composition of intestinal microbiota, alleviates intestinal inflammation, further improve the efficacy of chemotherapy which may contribute to the remission of CINV (26, 51). Meng et al. discovered that *L. plantarum* orally administration significantly increased the abundance of *Lactobacillus and Bifidobacterium* in cecal content of CTX-treated mice, and markedly reduced the nausea and vomiting symptoms, suggesting that the high abundance of *Lactobacillus* and *Bifidobacterium* is positively correlated with the relief of nausea, vomiting (52). What’s more, the increased abundance of *Lactobacillu*s limits intestinal tumor growth *via* gut microbiota reconstruction, tumor cell proliferation suppression and apoptosis activation (53, 54). *Bifidobacterium*, one of the butyrate-producing bacteria, has similarly been verified effective in decreasing pro-inflammatory cytokines and inhibiting cancer cells to suppress gastrointestinal cancer (55, 56). Notably, *Ruminococcus*, another strain can produce butyrate, showed a slight increase in abundance after *C. butyricum* administration. And there are published papers supporting that *Ruminococcus* could partially inhibit inflammation and protect the intestinal mucosa after cisplatin therapy in various cancer models (51, 57). At the phylum level, the findings of Burkhardt Flemer et al. (58) and our research both pointed to the decreased abundance of Firmicutes in CRC mice (**Figure 4A**). As suggested by another study, the diminished Firmicutes would be the possible mechanism responsible for cisplatin-associated side effects (47). In conclusion, the improved gut microbiota dysbiosis mediated by *C. butyricum* administration may be associated with anti-tumor, neurotransmitter secretion decrease and brain activation reduction, as well as anti-inflammation in colon.

In recent years, evidence has been mounting to suggest that chronic inflammation produces considerable inflammatory mediators (IL-6, IL-1β, TNF-α), activating NF-κB, thus leading to intestinal barrier function loss and colon carcinogenesis (59). Hence, we utilized H&E staining to find that the combination therapy (MCSL group) greatly suppresses colonic inflammation and attenuates histopathological changes in colon (**Figures 5A–B**). Additionally, our findings suggested that the combined treatment of THD and *C. butyricum* degraded the levels of IL-6, IL-1β, and TNF-α and downregulated the activity of HDAC1, TLR4, MyD88, and p-p65 (**Figures 5C–J**). Among them, histone deacetylase 1 (HDAC1) can be inhibited by butyrate to eliminate inflammation in colonic epithelial cells, subsequently suppressing colonic inflammation (60). The intestinal mucosal barrier functions are associated with intestinal tight-junction associated proteins, such as occludin (61). Besides, recent reports have suggested the importance of Trek1 in preserving the integrity of the intestinal epithelial barrier (62). We discovered that occludin and Trek1 activity in colon was significantly decreased in both M and MC groups. However, combination administration (MCSL group) substantially restored those reductions (**Figures 5K–M**). Collectively, these outcomes demonstrated that the combination of *C. butyricum* and THD has a notable impact on protecting intestinal barrier function and anti-inflammatory effect in CRC mice undergoing cisplatin chemotherapy.

## 5 Conclusions

**Figure 6:**
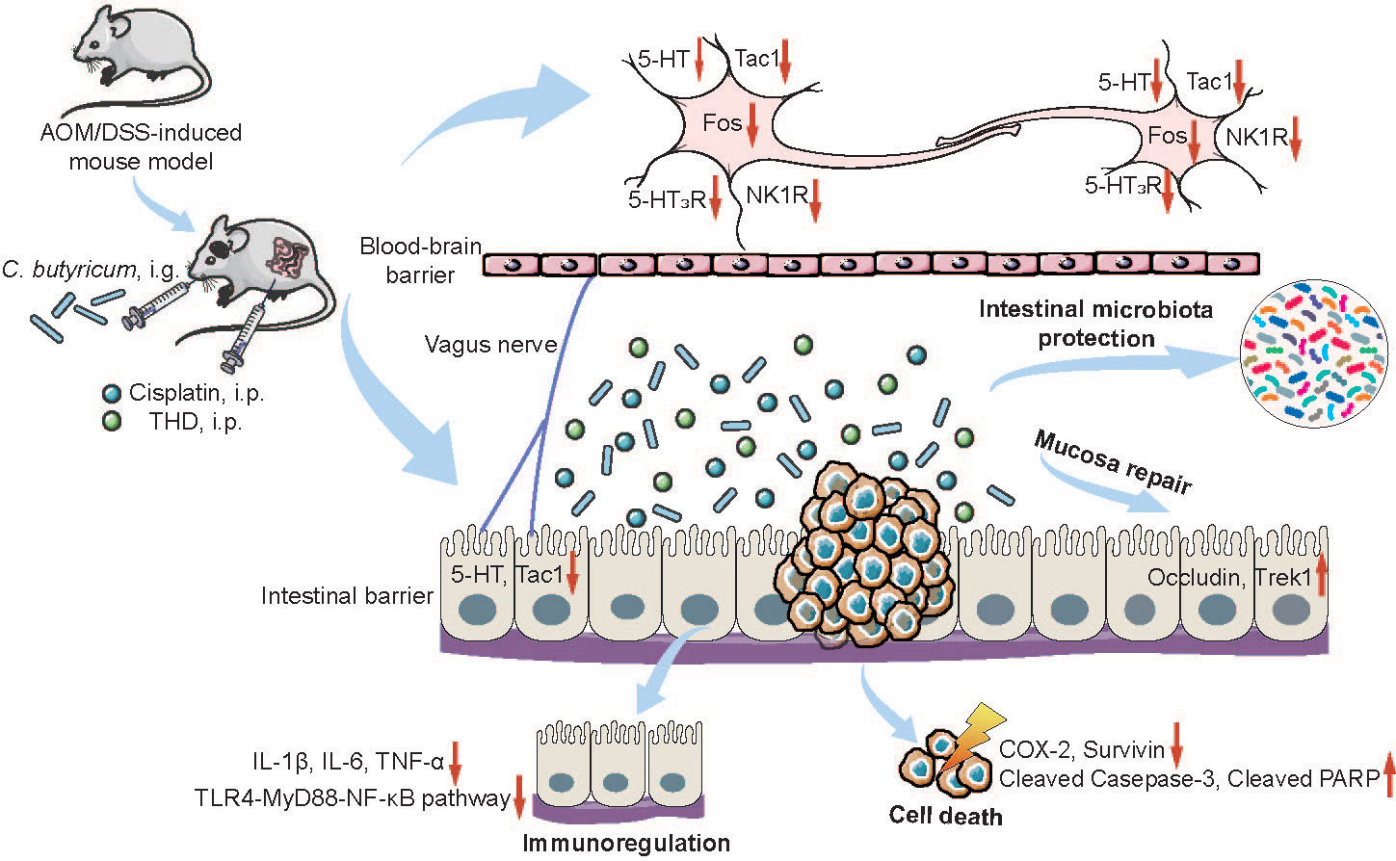
Schematic diagram of the underlying mechanisms of the combination of THD and *C. butyricum* in cancer treatments and CINV amelioration. The combination of THD and *C. butyricum* enhanced anti-tumor effects and ameliorated CINV *via* activating caspase-3 apoptosis pathway, inhibiting neurotransmitter (e.g., 5-HT and Tac1) and its receptors (e.g., 5-HT_3_R and NK-1R), and reversing intestinal dysbiosis in Azoxymethane/Dextran Sodium Sulfate induced colorectal cancer (CRC) mice. THD, thalidomide; *C. butyricum*, *Clostridium butyricum*; CINV, chemotherapy-induced nausea and vomiting; 5-HT, 5-hydroxytryptamine; Tac1, tachykinin 1; 5-HT_3_R, 5-hydroxytryptamine-3 receptor; NK1R, neurokinin-1 receptor.

We conclude that the combination of THD and *C. butyricum* improved cancer suppression efficacy of cisplatin in CRC mice by activation of caspase-3 apoptotic pathway. Moreover, our study confirmed that the inhibition of neurotransmitters (e.g., 5-HT and Tac1) and its receptor (e.g., 5-HT_3_R and NK-1R), the regulation of intestinal microbiota, the inhibition of inflammation in colon, and the protection of intestinal mucosa barrier *via the* combination therapy are extremely important to alleviate CINV in CRC mice (**Figure 6**). It should be noted that in this work, we only investigated the ability of *C. butyricum* combined with THD in the treatment of CRC and CINV *via* gut microbiota and vagus nerve activity modulation. However, it is important to evaluate the efficacy of additional probiotics for the development of chemotherapy adjuvant drugs. Moreover, we will fully consider the necessary of verifying the efficacy of the combination of *C. butyricum* and THD in CRC patients receiving chemotherapy and its chemotherapy associated side effects in the clinic.

## Data availability statement

The datasets presented in this study can be found in online repositories. The names of the repository/repositories and accession number(s) can be found below: https://www.ncbi.nlm.nih.gov/, PRJNA941024.

## Ethics statement

This study and the animal experimental protocol were reviewed and approved by the Laboratory Animal Ethics Committee of Nanchang Leyou Biotechnology Co. Ltd. (RyE2021070902). All experiments were conducted in accordance with the guidelines.

## Author contributions

XZ contributed with methodology, investigation, formal analysis, visualization and writing - original draft. HWu and RZ contributed with methodology, investigation and visualization. GS and JW contributed with investigation. HS and PT contributed with methodology. TC and HWe contributed with conceptualization, funding acquisition, supervision, writing – review and editing. All authors contributed to the article and approved the submitted version.

## Supplementary material

Supplementary Figure 1 The combination of THD and *C. butyricum* promote PARP cleaved in CRC mice. **(A)** Western blotting of cleaved PARP (n=3). **(B)** Relative expressions of cleaved PARP (n=3). Significance determined using one-way ANOVA with Tukey’s multiple comparison test and expressed as mean ± SD, * *P* < 0.05, ** *P* < 0.01.

## References

[1] Mao W Fan Y Cheng C Yuan X Lan T Mao K. Efficacy and safety of kanglaite injection combined with chemotherapy for colorectal cancer: a protocol for systematic review and meta-analysis. Med (Baltimore) (2020) 99:e22357. doi: 10.1097/MD.0000000000022357 PMC7523838 32991451

[2] Ren C Han H Pan J Chang Q Wang W Guo X. DLGAP1 − AS2 promotes human colorectal cancer progression through trans − activation of Myc. Mamm Genome (2022) 33:672–83. doi: 10.1007/s00335-022-09963-y 36222892

[3] Miller KD Nogueira L Devasia T Mariotto AB Yabroff KR Jemal A. Cancer treatment and survivorship statistics, 2022. CA Cancer J Clin (2022) 72:409–36. doi: 10.3322/caac.21731 35736631

[4] Oun R Moussa YE Wheate NJ. The side effects of platinum-based chemotherapy drugs: a review for chemists. Dalt Trans (2018) 47:6645–53. doi: 10.1039/c8dt00838h 29632935

[5] Yang Y Zhao Y Liu L Zhu W Jia S Li X. The anti-apoptotic role of COX-2 during *In vitro* infection of human intestinal cell line by giardia duodenalis and the potential regulators. Infect Immun (2022) 90:1–11. doi: 10.1128/iai.00672-21 PMC8929382 35130451

[6] Florea AM Büsselberg D. Cisplatin as an anti-tumor drug: cellular mechanisms of activity, drug resistance and induced side effects. Cancers (Basel) (2011) 3:1351–71. doi: 10.3390/cancers3011351 PMC3756417 24212665

[7] Rapoport BL. Delayed chemotherapy-induced nausea and vomiting: pathogenesis, incidence, and current management. Front Pharmacol (2017) 8:19. doi: 10.3389/fphar.2017.00019 28194109 PMC5277198

[8] Liu Y Zhang J Teng Y Zhang L Yu P Jin B. Thalidomide improves prevention of chemotherapy-induced gastrointestinal side effects following a modified FOLFOX7 regimen: results of a prospective randomized crossover study. Tumori (2009) 95:691–6. doi: 10.1177/030089160909500609 20210231

[9] Rolnick SJ Pawloski PA Hedblom BD Asche SE Bruzek RJ. Patient characteristics associated with medication adherence. Clin Med Res (2013) 11:54–65. doi: 10.3121/cmr.2013.1113 23580788 PMC3692389

[10] Hornby PJ. Central neurocircuitry associated with emesis. Am J Med (Elsevier Inc.) (2001), 106–12. doi: 10.1016/s0002-9343(01)00849-x 11749934

[11] Minami M Endo T Hirafuji M Hamaue N Liu Y Hiroshige T. Pharmacological aspects of anticancer drug-induced emesis with emphasis on serotonin release and vagal nerve activity. Pharmacol Ther (2003) 99:149–65. doi: 10.1016/S0163-7258(03)00057-3 12888110

[12] Rao KV Faso A. Chemotherapy-induced nausea and vomiting: optimizing prevention and management. Am Heal Drug Benefits (2012) 5:232–40. PMC4046471 24991322

[13] Navari RM. Management of chemotherapy-induced nausea and vomiting in pediatric patients. Pediatr Drugs (2017) 19:213–22. doi: 10.1007/s40272-017-0228-2 28447301

[14] J.D’Amato R S.Loughnan M Flynn E Folkman J. Thalidomide is an inhibitor of angiogenesis. Med Sci (1994) 91:4082–5. doi: 10.1073/pnas.91.9.4082 PMC43727 7513432

[15] Kenyon BM Browne F D’Amato RJ. Effects of thalidomide and related metabolites in a mouse corneal model of neovascularization. Exp Eye Res (1997) 64:971–8. doi: 10.1006/exer.1997.0292 9301478

[16] Wang N Xu P Liu Y Zhao P Ruan J Zheng Y. Efficacy and safety of thalidomide for chemotherapy-induced nausea and vomiting. J Cancer (2020) 11:4560–70. doi: 10.7150/jca.45678 PMC7255358 32489473

[17] Zhang L Qu X Teng Y Shi J Yu P Sun T. Efficacy of thalidomide in preventing delayed nausea and vomiting induced by highly emetogenic chemotherapy: a randomized, multicenter, double-blind, placebo-controlled phase III trial (CLOG1302 study). J Clin Oncol (2017) 35:3558–65. doi: 10.1200/JCO.2017.72.2538 28854065

[18] Wang X Shen Y Li S Lv M Zhang X Yeng J. Importance of the interaction between immune cells and tumor vasculature mediated by thalidomide in cancer treatment (Review). Int J Mol Med (2016) 38:1021–9. doi: 10.3892/ijmm.2016.2724 27599781

[19] Hayashi T Hideshima T Akiyama M Podar K Yasui H Rajen N. Molecular mechanisms whereby immunomodulatory drugs activate natural killer cells: Clinical application. Br J Haematol (2005) 128:192–203. doi: 10.1111/j.1365-2141.2004.05286.x 15638853

[20] Chen HM Yu YN Wang JL Lin YW Kong X Yang CQ. Decreased dietary fiber intake and structural alteration of gut microbiota in patients with advanced colorectal adenoma. Am J Clin Nutr (2013) 97:1044–52. doi: 10.3945/ajcn.112.046607 23553152

[21] Yixia Y Sripetchwandee J Chattipakorn N Chattipakorn SC. The alterations of microbiota and pathological conditions in the gut of patients with colorectal cancer undergoing chemotherapy. Anaerobe (2021) 68:102361. doi: 10.1016/j.anaerobe.2021.102361 33781900

[22] Cool RM Herrington JD. Thalidomide for the treatment of relapsed and refractory multiple myeloma. Pharmacotherapy (2002) 22:1019–28. doi: 10.1592/phco.22.12.1019.33606 12173786

[23] Meng C Bai C Brown TD Hood LE Tian Q. Human gut microbiota and gastrointestinal cancer. Genomics Proteomics Bioinforma (2018) 16:33–49. doi: 10.1016/j.gpb.2017.06.002 PMC6000254 29474889

[24] Yang N Zhan Y Wan J Li Y Hu X Liu W. Effects of lacidophilin tablets, yogurt, and bifid triple viable capsules on the gut microbiota of mice with antibiotic-associated diarrhea. Can J Infect Dis Med Microbiol (2022) 2022:1–10. doi: 10.1155/2022/6521793 PMC8964159 35360462

[25] Aragon G Graham DB Borum M Doman DB. Probiotic therapy for irritable bowel syndrome. Gastroenterol Hepatol (2010) 6:39–44. PMC2886445 20567539

[26] Stoeva MK Garcia-So J Justice N Myers J Tyagi S Nemchek M. Butyrate-producing human gut symbiont, clostridium butyricum, and its role in health and disease. Gut Microbes (2021) 13:1–28. doi: 10.1080/19490976.2021.1907272 PMC8078720 33874858

[27] Koh A De Vadder F Kovatcheva-Datchary P Bäckhed F. From dietary fiber to host physiology: short-chain fatty acids as key bacterial metabolites. Cell (2016) 165:1332–45. doi: 10.1016/j.cell.2016.05.041 27259147

[28] Hagihara M Kuroki Y Ariyoshi T Higashi S Fukuda K Yamashita R. Clostridium butyricum modulates the microbiome to protect intestinal barrier function in mice with antibiotic-induced dysbiosis. iScience (2020) 23:100772. doi: 10.1016/j.isci.2019.100772 PMC6970176 31954979

[29] Chen D Jin D Huang S Wu J Xu M Liu T. Clostridium butyricum, a butyrate-producing probiotic, inhibits intestinal tumor development through modulating wnt signaling and gut microbiota. Cancer Lett (2020) 469:456–67. doi: 10.1016/j.canlet.2019.11.019 31734354

[30] Liu J Sun J Wang F Yu X Ling Z Li H. Neuroprotective effects of clostridium butyricum against vascular dementia in mice *via* metabolic butyrate. BioMed Res Int (2015) 2015:1–12. doi: 10.1155/2015/412946 PMC4615854 26523278

[31] Gupta K Walton R Kataria SP. Chemotherapy-induced nausea and vomiting: pathogenesis, recommendations, and new trends. Cancer Treat Res Commun (2021) 26:100278. doi: 10.1016/j.ctarc.2020.100278 33360668

[32] Aapro M. CINV: still troubling patients after all these years. Support Care Cancer (2018) 26:5–9. doi: 10.1007/s00520-018-4131-3 29556808 PMC5876280

[33] Wang Y Li H. Gut microbiota modulation: a tool for the management of colorectal cancer. J Transl Med (2022) 20:1–14. doi: 10.1186/s12967-022-03378-8 35449107 PMC9022293

[34] Zou YT Zhou J Wu CY Zhang W Shen H Xu JD. Protective effects of poria cocos and its components against cisplatin-induced intestinal injury. J Ethnopharmacol (2021) 269:113722. doi: 10.1016/j.jep.2020.113722 33352240

[35] Kim J Lee HK. Potential role of the gut microbiome in colorectal cancer progression. Front Immunol (2022) 12:807648. doi: 10.3389/fimmu.2021.807648 35069592 PMC8777015

[36] Konturek PC Rembiasz K Burnat G Konturek SJ Tusinela M Bielanski W. Effects of cyclooxygenase-2 inhibition on serum and tumor gastrins and expression of apoptosis-related proteins in colorectal cancer. Dig Dis Sci (2006) 51:779–87. doi: 10.1007/s10620-006-3206-z 16615003

[37] Choi J Chang HK. The expression of MAGE and SSX, and correlation of COX2, VEGF, and survivin in colorectal cancer. Anticancer Res (2012) 32:559–64. 22287745

[38] Temraz S Mukherji D Shamseddine A. Potential targets for colorectal cancer prevention. Int J Mol Sci (2013) 14:17279–303. doi: 10.3390/ijms140917279 PMC3794728 23975167

[39] Wu F-H Wei H-Z Deng H-Y Xiao G-H Zhang Y-C. PARP in colorectal cancer: molecular mechanisms, immunity, clinical trials, and drug combinations. Neoplasma (2023) 70:1–14. doi: 10.4149/neo_2022_220724N745 36129834

[40] Han ZX Xu J Wang HM Ma J Sun X Du XP. Antiemetic role of thalidomide in a rat model of cisplatin-induced emesis. Cell Biochem Biophys (2014) 70:361–5. doi: 10.1007/s12013-014-9921-8 24718779

[41] Goineau S. Comparison of three preclinical models for nausea and vomiting assessment. J Pharmacol Toxicol Methods (2016) 82:45–53. doi: 10.1016/J.VASCN.2016.07.006 27477617

[42] Bullitt E. Expression of c-fos-like protein as a marker for neuronal activity following noxious stimulation in the rat. J Comp Neurol (1990) 296:517–30. doi: 10.1002/cne.902960402 2113539

[43] Li S Lei Y Chen JDZ. Chemotherapy-induced pica in rats reduced by electroacupuncture. Neuromodulation (2018) 21:254–60. doi: 10.1111/ner.12712 PMC6946630 29094451

[44] Marx W Ried K McCarthy AL Vitetta L Sali A McKavanagh D. Ginger–mechanism of action in chemotherapy-induced nausea and vomiting: a review. Crit Rev Food Sci Nutr (2017) 57:141–6. doi: 10.1080/10408398.2013.865590 25848702

[45] Li X Wu J Kang Y Chen D Chen G Zeng X. Yeast mannoproteins are expected to be a novel potential functional food for attenuation of obesity and modulation of gut microbiota. Front Nutr (2022) 9:1019344. doi: 10.3389/fnut.2022.1019344 36313084 PMC9614242

[46] Fong W Li Q Yu J. Gut microbiota modulation: a novel strategy for prevention and treatment of colorectal cancer. Oncogene (2020) 39:4925–43. doi: 10.1038/s41388-020-1341-1 PMC7314664 32514151

[47] Gori S Inno A Belluomini L Bocus P Bisoffi Z Russo A. Gut microbiota and cancer: how gut microbiota modulates activity, efficacy and toxicity of antitumoral therapy. Crit Rev Oncol Hematol (2019) 143:139–47. doi: 10.1016/j.critrevonc.2019.09.003 31634731

[48] Chen X Yi H Liu S Zhang Y Su Y Liu X. Probiotics improve eating disorders in mandarin fish (Siniperca chuatsi) induced by a pellet feed diet *via* stimulating immunity and regulating gut microbiota. Microorganisms (2021) 9:1288. doi: 10.3390/microorganisms9061288 PMC8231599 34204793

[49] Wang H Chen G Li X Zheng F Zeng X. Yeast β-glucan, a potential prebiotic, showed a similar probiotic activity to inulin. Food Funct (2020) 11:10386–96. doi: 10.1039/D0FO02224A 33231600

[50] Luo X Kong Q Wang Y Duan X Wang P Li C. Colonization of clostridium butyricum in rats and its effect on intestinal microbial composition. Microorganisms (2021) 9:1573. doi: 10.3390/microorganisms9081573 PMC8401576 34442652

[51] Hsiao Y-P Chen H-L Tsai J-N Lin M-Y Liao J-W. Administration of lactobacillus reuteri combined with clostridium butyricum attenuates cisplatin-induced renal damage by gut microbiota reconstitution, increasing butyric acid production, and suppressing renal inflammation. Nutrients (2021) 13:2792. doi: 10.3390/nu13082792 PMC8402234 34444952

[52] Meng Y Wang J Wang Z Zhang G Liu L Huo G. Lactobacillus plantarum KLDS1.0318 ameliorates impaired intestinal immunity and metabolic disorders in cyclophosphamide-treated mice. Front Microbiol (2019) 10:731. doi: 10.3389/fmicb.2019.00731 31031723 PMC6473033

[53] Sugimura N Li Q Chu ESH Lau HCH Fong W Liu W. Lactobacillus gallinarum modulates the gut microbiota and produces anti-cancer metabolites to protect against colorectal tumourigenesis. Gut (2022) 71:2011–21. doi: 10.1136/gutjnl-2020-323951 PMC9484392 34937766

[54] Dong Y Zhu J Zhang M Ge S Zhao L. Probiotic lactobacillus salivarius ren prevent dimethylhydrazine-induced colorectal cancer through protein kinase b inhibition. Appl Microbiol Biotechnol (2020) 104:7377–89. doi: 10.1007/s00253-020-10775-w 32666185

[55] Zaharuddin L Mokhtar NM Najmi K Nawawi M Affendi R Ali R. A randomized double-blind placebo- controlled trial of probiotics in post-surgical colorectal cancer. (2019) 19:131. doi: 10.1186/s12876-019-1047-4 PMC6657028 31340751

[56] Bahmani S Azarpira N Moazamian E. Anti-colon cancer activity of bifidobacterium metabolites on colon cancer cell line SW742. Turkish J Gastroenterol (2019) 30:835–42. doi: 10.5152/tjg.2019.18451 PMC6750814 31530527

[57] Perales-Puchalt A Perez-Sanz J Payne KK Svoronos N Allegrezza MJ Chaurio RA. Frontline science: microbiota reconstitution restores intestinal integrity after cisplatin therapy. J Leukoc Biol (2018) 103:799–805. doi: 10.1002/JLB.5HI1117-446RR 29537705 PMC6004318

[58] Flemer B Lynch DB Brown JMR Jeffery IB Ryan FJ Claesson MJ. Tumour-associated and non-tumour-associated microbiota in colorectal cancer. Gut (2017) 66:633–43. doi: 10.1136/gutjnl-2015-309595 PMC5529966 26992426

[59] Saleh M Trinchieri G. Innate immune mechanisms of colitis and colitis-associated colorectal cancer. Nat Rev Immunol (2011) 11:9–20. doi: 10.1038/nri2891 21151034

[60] Zimmerman MA Singh N Martin PM Thangaraju M Ganapathy V Waller JL. Butyrate suppresses colonic inflammation through HDAC1-dependent fas upregulation and fas-mediated apoptosis of T cells. Am J Physiol - Gastrointest Liver Physiol (2012) 302:1405–15. doi: 10.1152/ajpgi.00543.2011 PMC3378095 22517765

[61] Fanning AS Jameson BJ Jesaitis LA Anderson JM. The tight junction protein ZO-1 establishes a link between the transmembrane protein occludin and the actin cytoskeleton. J Biol Chem (1998) 273:29745–53. doi: 10.1074/jbc.273.45.29745 9792688

[62] Huang H Liu JQ Yu Y Mo LH Ge RT Zhang HP. Regulation of TWIK-related potassium channel-1 (Trek1) restitutes intestinal epithelial barrier function. Cell Mol Immunol (2016) 13:110–8. doi: 10.1038/cmi.2014.137 PMC4711681 25683610

