# Supplementary material for: Combination of thalidomide and Clostridium butyricum relieves chemotherapy-induced nausea and vomiting via gut microbiota and vagus nerve activity modulation
Source: Front Immunol. 2023 Jun 22;14:1220165. doi: 10.3389/fimmu.2023.1220165 (PMC10327820; doi:10.3389/fimmu.2023.1220165)
Supplement: Supplementary file 1 [file Table_1.docx]

**Supplementary Material Table S1**

| qPCR target | Forward 5’-3’ | Reverse5’-3’ |
| --- | --- | --- |
| Il1b | GTGTCTTTCCCGTGGACCTTC | TCATCTCGGAGCCTGTAGTGC |
| Tnf | GTGGAACTGGCAGAAGAGGCA | AGAGGGAGGCCATTTGGGAAC |
| Il6 | CTTCTTGGGACTGATGCTGGTGAC | AGGTCTGTTGGGAGTGGTATCCT |
| Gapdh | CTCGTGGAGTCTACTGGTGT | GTCATCATACTTGGCAGGTT |
| Tac1 | GCCCTGTTAAAGGCTCTTTATG | CTTCTTTCGTAGTTCTGCATCG |
