# Supplementary material for: Combination of thalidomide and Clostridium butyricum relieves chemotherapy-induced nausea and vomiting via gut microbiota and vagus nerve activity modulation
Source: Front Immunol. 2023 Jun 22;14:1220165. doi: 10.3389/fimmu.2023.1220165 (PMC10327820; doi:10.3389/fimmu.2023.1220165)
Supplement: Supplementary file 2 [file Table_2.docx]

**Supplementary Material Table S2**

| Antibodies | Source | Cat. No. |
| --- | --- | --- |
| GAPDH | CST | 5174s |
| TLR4 | CST | 14358s |
| p-p65 | CST | 3033S |
| p65 | CST | 8242s |
| MyD88 | CST | 4283S |
| Occludin | Proteintech | 66378-1-Ig |
| Survivin | Proteintech | 10508-1-AP |
| Cleaved-PARP | Affinity | AF7023 |
| HDAC1 | Affinity | AF6433 |
| Trek1 | BioVision | 2841-100 |
| Cleaved-Caspase-3 | Affinity | AF7022 |
| COX-2 | Affinity | AF7003 |
| β-actin | CST | 4970S |
| c-Fos | Servicebio | GB12069 |
| 5HT3A receptor | Affinity | DF7437 |
| NK1R | Affinity | DF4996 |
